# Small Neuron-Derived Extracellular Vesicles from Individuals with Down Syndrome Propagate Tau Pathology in the Wildtype Mouse Brain

**DOI:** 10.3390/jcm10173931

**Published:** 2021-08-31

**Authors:** Aurélie Ledreux, Sarah Thomas, Eric D. Hamlett, Camille Trautman, Anah Gilmore, Emily Rickman Hager, Daniel A. Paredes, Martin Margittai, Juan Fortea, Ann-Charlotte Granholm

**Affiliations:** 1 Knoebel Institute for Healthy Aging, University of Denver, Denver, CO 80208, USA; sarahjthomas123@gmail.com (S.T.); cwtrautman@gmail.com (C.T.); anah.gilmore@du.edu (A.G.); daniel.paredes@du.edu (D.A.P.); lotta.granholm-bentley@du.edu (A.-C.G.); 2 Department of Pathology and Laboratory Medicine, Medical University of South Carolina, Charleston, SC 29425, USA; hamlette@musc.edu; 3 Department of Chemistry and Biochemistry, University of Denver, Denver, CO 80208, USA; emilyh108@yahoo.com (E.R.H.); martin.margittai@du.edu (M.M.); 4 Hospital de la Santa Creu i Sant Pau and Catalan Down Syndrome Foundation, 08041 Barcelona, Spain; JFortea@santpau.cat

**Keywords:** Down syndrome, aging, biomarkers, neuropathology, Alzheimer’s disease

## Abstract

Individuals with Down syndrome (DS) exhibit Alzheimer’s disease (AD) pathology at a young age, including amyloid plaques and neurofibrillary tangles (NFTs). Tau pathology can spread via extracellular vesicles, such as exosomes. The cargo of neuron-derived small extracellular vesicles (NDEVs) from individuals with DS contains p-Tau at an early age. The goal of the study was to investigate whether NDEVs isolated from the blood of individuals with DS can spread Tau pathology in the brain of wildtype mice. We purified NDEVs from the plasma of patients with DS-AD and controls and injected small quantities using stereotaxic surgery into the dorsal hippocampus of adult wildtype mice. Seeding competent Tau conformers were amplified in vitro from DS-AD NDEVs but not NDEVs from controls. One month or 4 months post-injection, we examined Tau pathology in mouse brains. We found abundant p-Tau immunostaining in the hippocampus of the mice injected with DS-AD NDEVs compared to injections of age-matched control NDEVs. Double labeling with neuronal and glial markers showed that p-Tau staining was largely found in neurons and, to a lesser extent, in glial cells and that p-Tau immunostaining was spreading along the corpus callosum and the medio-lateral axis of the hippocampus. These studies demonstrate that NDEVs from DS-AD patients exhibit Tau seeding capacity and give rise to tangle-like intracellular inclusions.

## 1. Introduction

Down syndrome (DS) is the most common aneuploidy and cause of intellectual disability of genetic origin, with an incidence of 1 in 600–700 babies [1]. The presence of a third copy of a segment of or the entire chromosome 21 (Hsa21) results in the overexpression of several genes, including the amyloid precursor protein (*APP*) gene. Due to the overexpression of several different genes in Hsa21, individuals with DS develop Alzheimer’s disease (AD) pathology, including neuroinflammation, neuronal cell loss, amyloid plaques and neurofibrillary tangles (NFTs) [2,3,4,5,6,7]. The prevalence of AD in DS (DS-AD) is significantly higher than that seen in the general population and occurs at a much earlier age [8]. The presence of the amyloid precursor protein (*APP)* gene on Hsa21 plays a role in this early onset AD [4,9], but other genes on this chromosome (e.g., RCAN1 and Dyrk1A) might also play a role in the development of AD pathology and clinical manifestations of dementia [10,11,12]. Pathological alterations of the microtubule-associated protein Tau occurs early in DS [5,13], leading to the intracellular accumulation of phosphorylated forms of Tau (p-Tau) into neurofibrillary tangles (NFTs) [6,14]. 

The Tau protein is a neuronal protein important for microtubule polymerization and stabilization [15,16]. The adult human brain has six Tau isoforms that all derive from one gene (*MAPT*) by alternative splicing of exons 2, 3 and 10 [17,18,19,20]. Under normal physiological conditions, there is a balance between Tau phosphorylation and dephosphorylation. The abnormal hyperphosphorylation of Tau contributes greatly to the conversion of normal Tau into paired helical filaments (PHFs) [17,21]. When Tau is hyperphosphorylated, it loses biological activity and becomes resistant to degradation, leading to conformational changes that render Tau insoluble and prone to aggregation [20,22,23,24,25,26]. Tau pathology plays a central role in several different neurodegenerative diseases including AD [20,27], DS-AD [5,13,14], frontotemporal dementia (FTD) [20,28] and chronic traumatic encephalopathy (CTE) [17,29,30,31,32,33]. The complicated seeding capacity of toxic Tau species has not yet been explored in depth but could provide a viable theory as to why AD and other tauopathies start in one part of the brain and spread in a network-like manner to other brain areas [34]. P-Tau pathology has been suggested to spread by a prion-like mechanism in the brain [35,36], and toxic forms of Tau have been shown to spread trans-synaptically [28,37]. Investigators have reported the seeding and spreading of human p-Tau into the brain of wildtype (WT) mice from brain homogenates prepared from individuals with various forms of tauopathies and have shown transfer of p-Tau pathology into neurons and, to some extent, also glial cells [16,18,27,35,36,38,39]. Ferrer and collaborators reported that injected p-Tau could spread to the ipsilateral hippocampus, corpus callosum and as far as the septal nuclei and the contralateral corpus callosum in adult WT mice [35]. Albert and colleagues have shown that the prion-like seeding and spreading of toxic p-Tau in the brain of WT mice could be blocked by a Tau neutralizing antibody, Tau antibody D, which recognizes an epitope in the central region of Tau [40]. They found that the Tau antibody D could not only neutralize the pathological species of Tau, but also prevent its spread to distant brain regions following injection [40]. However, biological mechanisms for the seeding of p-Tau from extracellular vesicles (EVs) have not been explored in depth.

Recently, EVs—including exosomes—have been recognized as carriers of AD-related pathological proteins as well as non-coding RNAs, lipids and surface markers [41,42,43,44]. There are at least the following three major types of EVs, based on their size, composition and biogenesis mechanisms: exosomes, microvesicles and apoptotic bodies [45,46]. Exosomes are a type of EV with a diameter of 40–150 nm and whose major functions include cell-cell communication and cell maintenance [46]. The biogenesis of exosomes occurs via the intracellular formation of multivesicular bodies (MVBs) and is regulated by two pathways—namely, the endosomal sorting complexes required for transport (ESCRT)-dependent or ESCRT-independent pathways [47]. The endosomal sorting machinery is important for the biogenesis and function of exosomes [41]. The vast differences in the contents of exosomal cargo and release mechanisms have been shown to be influenced by the cell of origin but also by disease conditions [48,49]. After being released from cells, small EVs and their cargo are taken up by other cells via interaction with various exosomal surface proteins and cellular receptors on the recipient cell membrane. Once attached, exosomes can be endocytosed, fused with the recipient cell and can promote intracellular signaling pathways, affecting gene expression, cell metabolism and other important functions of the recipient cell [50,51,52]. It is known that essentially all the cells of the CNS release exosomes, including neurons [30,53], astrocytes [54,55,56,57,58], microglia [54,59] and oligodendrocytes [60]. While some exosomes exert effects in their proximal milieu, others travel long distances in the brain to affect other cell types or networks [61]. Exosomes are involved in normal functions of the CNS such as cell-cell communication, transcription regulation, neurogenesis and plasticity, as well as the response to inflammation [62], but also neuron-glia interface [54], synaptic plasticity [63] and the regeneration/protection of neurons [64]. Exosomes have also been found to play a significant role in the transmission and propagation of protein aggregates associated with several neurodegenerative diseases [65,66,67].

Recently, multiple investigators have reported that exosomes isolated from AD plasma samples, transgenic Tau mouse brains or AD brain tissues can spread AD pathology, including Aβ and p-Tau, leading to cell damage and contribute to neuronal loss in AD [68,69,70,71,72]. Aβ peptides are present in exosome cargo, and these exosomes are accumulating around and within neuritic plaques in the brain of those with AD [65]. These findings suggest that exosomes, and potentially other EVs, can act as vehicles for the neuron-to-neuron or neuron-to-glia transfer of toxic species of amyloid and Tau in neurodegenerative disorders. On the other hand, exosomes can also exert a neuroprotective role in neurodegenerative disorders, for example, by reducing Aβ levels via the uptake of Aβ-containing exosomes secreted from neurons into microglia for amyloid metabolism [73], to spread growth factors to remote areas of the brain [74,75] or as a vehicles for the delivery of pharmacological therapies providing cell-free neurotherapy [76]. 

Based on the reported Tau alterations observed in individuals with DS [5,13,77,78,79], the current study was focused on the following two central questions: (1) Do neuron-derived EVs (NDEVs) isolated from the plasma of persons with DS-AD contain Tau species that have seeding capacity in the WT mouse brain, and (2) If so, to which cell types in the CNS do the toxic species of Tau spread? To answer these questions, we utilized several methods to characterize the NDEVs from controls and persons with DS-AD combined with stereotaxic injections into the WT mouse brain.

## 2. Materials and Methods

### 2.1. Isolation of NDEVs from Human Plasma

Plasma samples were obtained from persons with DS-AD or non-DS age-matched control participants via the Down Syndrome Biobank Consortium (DSBC) (study protocol approved by University of Denver Institutional Review Board). Plasma aliquots (0.25 mL) were treated with thrombin (System Biosciences, Palo Alto, CA, USA) for 30 min at room temperature, then mixed with 0.22 µm of filtered Dulbecco’s Phosphate Buffered Saline (DPBS) containing a protease and phosphatase inhibitor cocktail (Thermo Scientific, Inc., Waltham, MA, USA) [13,30,80]. After centrifugation, the supernatant was mixed with ExoQuick polymer solution (System Biosciences, CA, USA) and the resulting pellet was resuspended in ultra-pure H_2_O containing a protease and phosphatase inhibitor cocktail as previously described [13]. To selectively isolate neuronal EVs, samples were incubated for 1 h with mouse biotinylated anti-human CD171 monoclonal antibody (clone 5G3, L1CAM, eBiosciences, CA, USA). Then, Streptavidin-Plus Ultra-link beads (Thermo Scientific, Inc.) were added to each suspension and incubated for 4 h with gentle mixing. Immuno-absorbed samples were centrifuged at 400× *g* for 10 min, washed once with DPBS, then NDEVs were eluted with 0.1 M glycine-HCl (pH 3.0), incubated at 4 °C for 5 min and centrifuged for 5 min at 4500× *g* [13]. The supernatant was transferred to a fresh tube containing 1 M Tris-HCl and 3% BSA in DPBS. The volume was brought to 0.25 mL by adding DPBS, and NDEV preparations to be used for stereotaxic injections were stored at −80 °C until use for the intracranial surgeries. To determine the CD81, Tau and p-Tau levels in each exosome preparation, another aliquot of plasma was treated as described but resuspended in M-PER lysis buffer (ThermoFisher) instead of DPBS and underwent one freezing/thawing cycle. NDEVs were isolated from an initial volume of 0.25 mL of plasma and were resuspended in a final volume of 0.25 mL. Therefore, all the NDEV biomarker values refer to the original plasma volume. Levels of CD81 were measured using CD81 Elisa kit (Cusabio, Houston, TX, USA), while Tau (N-terminal to mid-domain (R1) Tau) and p-Tau T231 levels were measured using the Simoa SR-X instrument (Quanterix, MA, USA) according to the manufacturer’s instructions. 

### 2.2. Validation of NDEVs

NDEV size and concentration were assessed using nanoparticle tracking analysis with the Nanosight apparatus (Malvern Instruments). NDEVs were diluted in filtered DPBS and labeled with ExoGlow™ membrane labelling kit (System Biosciences, CA, USA) following manufacturer’s protocol. Particle enumeration and sizing were carried out on a LM10 Nanosight instrument on fluorescence mode. Analysis of particle counts was conducted with Nanosight NTA 2.3 software, using a detection threshold of five. The presence of exosomal markers, including the tetraspanins CD63 and CD81 was also confirmed using Western blotting [81] using Mini Protean TGX gels (Bio-Rad, Hercules, CA, USA). These were loaded with the maximum volume of NDEV per well and separated at 150 V for 40 min. Next, proteins were transferred to PVDF membrane. Membranes were probed overnight at 4 °C with antibodies against CD63 (EXOAB-CD63A-1, System Biosciences) and CD81 (SAB3500454, Sigma Aldrich), washed and incubated with appropriate secondary antibodies for 1 h at room temperature. Membranes were then washed and developed with SuperSignal™ West Femto chemiluminescent substrate (ThermoFisher, Hillsboro, OR, USA) and visualized on a ChemiDoc imaging system (Bio-Rad). 

**Figure 1:**
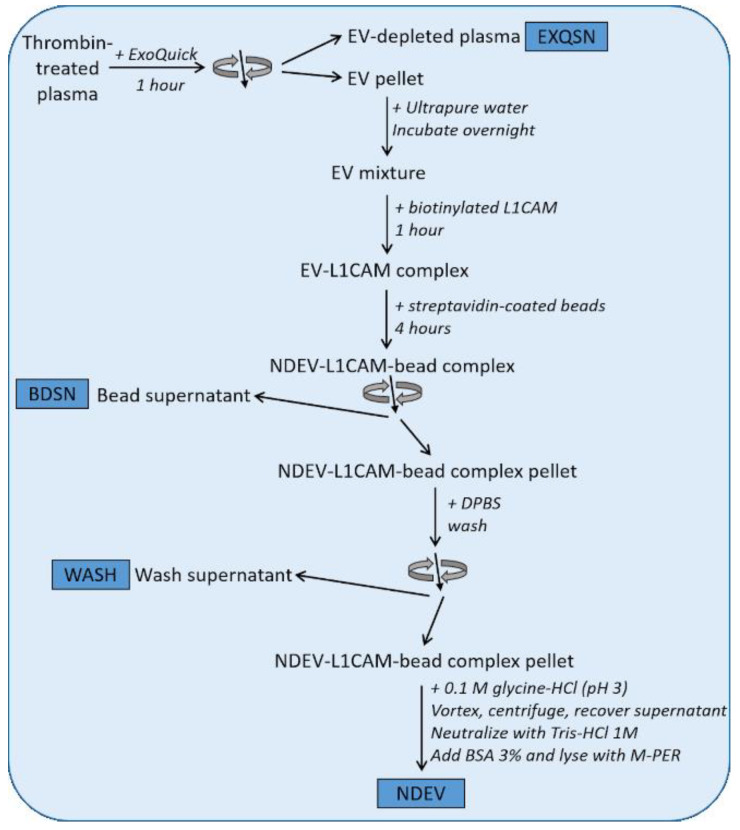
Experimental workflow for exosome validation studies. Thrombin-treated plasma was first processed with ExoQuick polymer reagent, whereafter the EV-depleted plasma (EXQSN) was collected. The EV pellets were then mixed with L1CAM biotinylated antibody, then streptavidin-coated beads. The bead supernatant (BDSN) was collected for biomarker analyses and the bead complex was next washed with Dulbecco’s Phosphate Buffered Saline (DPBS) (WASH was collected). Neuron-derived small extracellular vesicles (NDEVs) were then recovered from the mix. Exosome-related proteins and neuron-specific proteins were measured at each step of the isolation procedure. The results from these validation studies are presented in Figures 2 and 3.

To further demonstrate NDEV enrichment in our samples, we used four plasma samples from healthy volunteers and isolated NDEV as described above and, in addition to the final NDEV, collected supernatant after each step (EXQSN, WASH and BDSN) of the isolation protocol as depicted in Figure 1. EV-specific markers were measured using commercial ELISA kits: levels of membrane-bound CD81 and luminal Alix were assessed using ELISA kits (Cusabio, Houston, TX, USA). To demonstrate enrichment for neuron-specific proteins, we measured using single molecule array (Simoa) technology on the SR-X instrument (Quanterix, MA, USA) the levels of Tau, UCH-L1 and NF-light in plasma, the three different supernatants collected at each isolation step and the final NDEV preps (see experimental outline in Figure 1).

### 2.3. In Vitro Seeding and Conformational Tau Analysis

Seeding competent Tau conformers were amplified from NDEVs using a modified version of our previously published amplification protocol [24]. NDEVs from control and DS subjects were mixed with 0.1% Triton X-100, sonicated for 2 min on ice (tip sonicator) and then combined with recombinant Tau (hTau40) and heparin (Celsus, average molecular weight 4400 Da). Final volume was 700 μL, all mixtures contained 10 μM Tau, 40 μM heparin and NDEVs at a protein concentration of 150 μg/mL. The samples were subjected to 30 cycles of fracture and growth at 37 °C, utilizing a bath sonicator with a microplate horn (QSonica). Each cycle included a 5-second sonication step at 5% amplitude, followed by a 30-minute step of quiescent incubation. The amplified material was then sedimented by ultracentrifugation (30 min at 130,000× *g*). Pellets were analyzed using SDS-PAGE and Coomassie staining. 

### 2.4. Stereotaxic Surgery

At eight months of age, male C57BL/6 mice were anesthetized deeply with ketamine/xylazine (120 mg/kg/6 mg/kg) and immobilized in a mouse stereotaxic frame (Kopf Instruments) connected to a syringe pump system (Harvard Apparatus, Holliston, MA, USA). Mice were randomly assigned to be injected unilaterally with either 2 μL of NDEVs isolated from the blood of a DS-AD individual (*n* = 8) or 2 μL of NDEV from an age-matched control donor (*n* = 7). Burr holes were made using a Drexel drill and a 10-microliter Hamilton syringe was lowered to the dorsal layer of the hippocampus (AP −1.3 mm; ML 1.5 mm; DV −2.0 mm relative to Bregma) according to the Paxinos mouse atlas [82]. After the injection, the syringe was kept in place for 10 min to limit diffusion back into the needle track. The mice were maintained for either 1 month (*n* = 7) or 4 months (*n* = 8) after injection. All animal procedures followed the guidelines of the National Institutes of Health (NIH) Guide for the Care and Use of Laboratory Animals and were approved by the University of Denver Institutional Animal Care and Use Committee (IACUC).

### 2.5. Immunohistochemistry and Image Analysis

Mice were euthanized following NIH guidelines using carbon dioxide inhalation. Brains were rapidly removed and fixed for 48 h in 4% paraformaldehyde at 4 °C, then transferred to 30% sucrose solution in phosphate buffered saline (PBS, pH 7.4) for 1 week. Coronal sections (35-micron thickness) were obtained using a cryostat (Microm). Tau immunostaining was analyzed using two different commercial phospho-Tau antibodies. NFTs represent cytoplasmic inclusions of abnormal insoluble aggregates in the brain of patients with AD. These aggregates are paired helical filaments (PHFs), mostly consisting of Tau protein. Conformational changes of Tau involve hyperphosphorylation and truncation resulting in PHF assembly. In the current study, we examined different stages of Tau aggregation using two immunological markers of specific N-terminus phosphorylation Tau sites, including a marker for early stages of abnormal Tau processing, T231, and a marker for a later stage, S396 [83]. Coronal sections were blocked for 1 h with 10% normal serum, then permeabilized in 0.1% Triton-X in TBS. Sections were incubated overnight at 4 °C with primary antibodies against p-Tau S396 (Invitrogen, Cat# 44-752G) and P-Tau T231 (Invitrogen, Cat# 44-746G), washed and then incubated for 1 h with appropriate secondary antibodies (Alexa Fluor 594, Life Technologies, 1:250) at room temperature. The stained sections were washed and mounted using ProLong Gold antifade mounting media (Molecular Probes). Images were obtained with a laser scanning confocal microscope (Olympus FV3000 Laser Scanning Confocal microscope) using FluoView software (Olympus). The intensity of p-Tau immunofluorescence was estimated using ImageJ [84] by creating a maximum projection of all the slices in the stack, subtracting the background (rolling ball radius of 25 pixels) and measuring mean integrated density. Staining intensity was measured separately in the hilar region (polymorphic layer) and the granule cell layer of the dentate gyrus, on at least two different brain sections per animal, and averaged to obtain a single value for each antibody and animal.

Double immunofluorescence staining was performed with either p-Tau antibodies (S396 or T231, as described previously) and with GFAP (Glial fibrillary acidic protein, Dako, 1:100), Iba1 (ionized calcium-binding adapter molecule 1, Wako, 1:500) or NeuN (Chemicon, 1:200) to determine whether p-Tau co-localized with astrocytic, microglial or neuronal markers, respectively. Double immunofluorescent labeling was carried out according to our previously published protocols [85]. Sections were incubated overnight at 4 °C with p-Tau 396 or p-Tau T231 and antibodies against astrocytes, microglia or neural cell bodies as described above. p-Tau S396 and p-Tau T31 were visualized with Alexa Fluor 594-labelled secondary antibody (Life Technologies, Carlsbad, CA, USA, 1:250) and GFAP, Iba1 and NeuN were visualized using Alexa Fluor 488 (Life Technologies, 1:250). Mounted sections were imaged in stacks in both channels via laser scanning confocal microscope and processed as described above. The staining intensity for Iba1 and GFAP was determined as described above. The ImageJ Coloc2 plugin was used to determine the pixel intensity correlation in the dentate gyrus area between the two channels (using the same ROI for both channels). Scatter dot plots were generated by plotting the average Pearson’s pixel intensity correlation coefficient between p-Tau S396 or T231 and GFAP, NeuN or Iba1 for each mouse.

### 2.6. Statistical Analyses 

All data are presented as mean ± standard error of the mean (SEM). For each time point (1 month and 4 months post-injection), two-way ANOVA (treatment × injection side) were run to determine differences in staining intensities between the DS-AD- and control-injected groups. Statistical analyses were performed using GraphPad Prism version 8 (GraphPad Software, San Diego, CA, USA). A statistically significant difference was assumed at *p* < 0.05.

## 3. Results

### 3.1. NDEV Validation and Characterization

**Figure 2:**
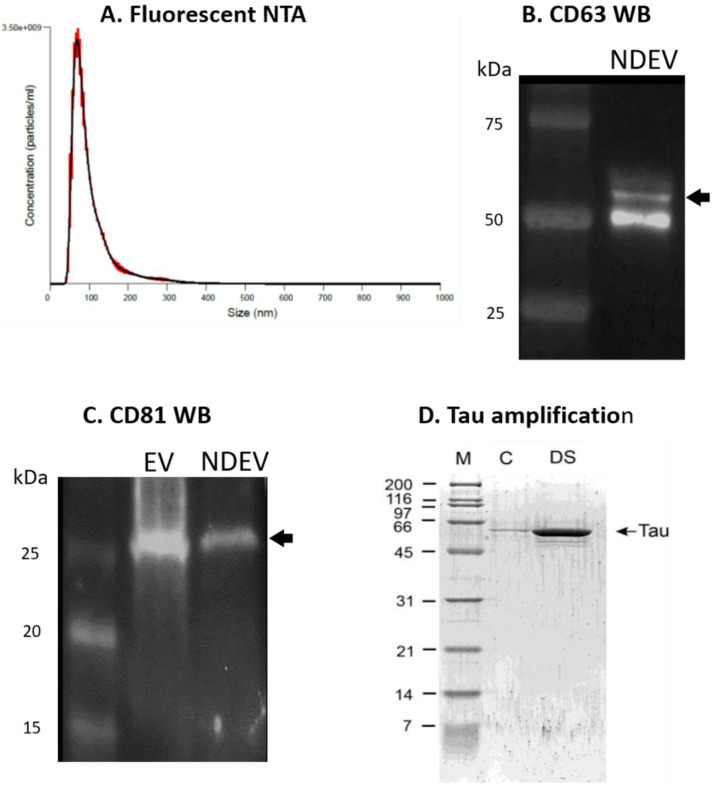
NDEV characterization. (**A**) Fluorescent nanoparticle tracking analysis illustrating size distribution and concentration of extracellular vesicles in neuron-derived exosome samples. The single peak at ≈100 nm suggests small extracellular vesicle (EV)-specific enrichment. (**B**) Representative Western blot image of CD63 tetraspanin protein indicative of EV enrichment. CD63 (System Biosciences, EXOAB-CD63A-1) was detected at 53 kD in NDEVs isolated from human plasma via Western blot. (**C**) Representative Western blot showing tetraspanin CD81 (SAB3500454, Sigma Aldrich) at 25 kD in total EVs (*EV*) from human plasma (System Biosciences, EXOP-500A-1) and neuron-derived EVs (*NDEV*) isolated from human plasma. (**D**) Amplification of Tau fibrils from NDEVs obtained from blood. M = marker, C = control NDEVs, DS = NDEVs from plasma from a subject with Down syndrome.

**Figure 3:**
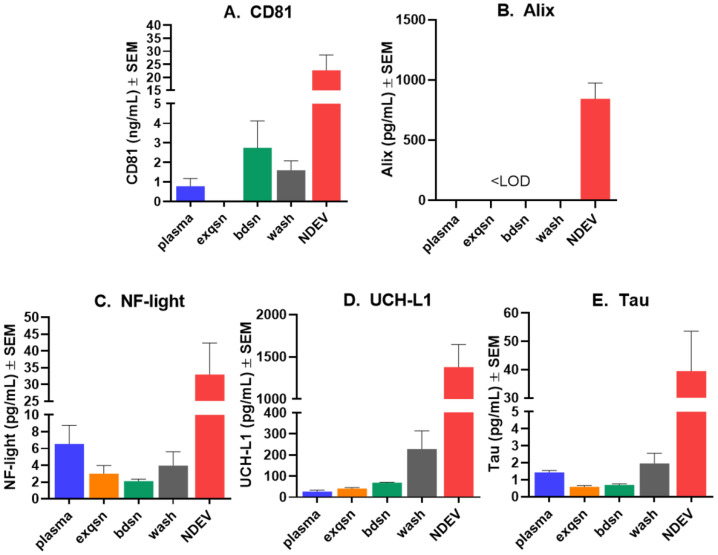
Quantification of biomarkers showing enrichment for neuron-derived EVs. Biomarkers were measured in plasma, EV-depleted plasma (exqsn), bead supernatant (bdsn), wash supernatant (wash) and in NDEVs (described in Figure 1). Levels of small EV-associated tetraspanin CD81 (**A**) and Alix (**B**) were measured using commercial ELISA kits (Cusabio). Levels of neuron-specific proteins NF-light (**C**), UCH-L1 (**D**) and Tau (**E**) in plasma were measured using Simoa assay kits on the SR-X instrument (Quanterix). Note that NDEVs contained significantly higher levels of all components including the neuron-specific markers, strongly suggesting that the immunoprecipitation procedure for NDEVs had succeeded. The same process was used to obtain the NDEVs for injection into mouse brain.

The analysis of NDEVs using fluorescent NTA revealed an enrichment in the expected size range (Figure 2A), with a concentration of ≈10^9^–10^10^ particles/mL and an average size of 107 ± 4.2 nm. The size distribution and particle concentration are within the range reported by other investigators [70,86,87]. By Western blotting, we found that the NDEV samples contained both CD63 and CD81, which are members of the tetraspanin family and are located on the exosome surface (Figure 2B,C). Using commercially available ELISA kits, we measured EV-specific markers CD81 and Alix levels in the plasma, NDEV preps as well as the three supernatants collected throughout the isolation procedure (see Figure 1 for experimental design). We found that the CD81 and Alix levels were significantly elevated in the NDEV preps compared to the plasma and to the three supernatants collected through the isolation procedure (Figure 3A,B), demonstrating a specific enrichment for EVs in our preparations. Next, to confirm the enrichment for neuronal EVs in our preparations, we measured the levels of relatively specific neuronal biomarkers (Tau, NF-light and UCH-L1) in the NDEV preps as well as in the matching plasma samples and in the three different supernatants recovered during the isolation process. All three proteins were measurable to some extent in the plasma samples using the Simoa technology (Quanterix, MA) (Figure 3C–E). However, when comparing the NDEV with the plasma levels, we observed a clear enrichment of these three neuronal proteins, with the NDEV levels being between nine and 50 times higher than the plasma levels, strongly suggesting that the NDEV enrichment protocol used herein successfully isolated neuron-derived EVs.

In the NDEV preps to be used for stereotactic injections, the levels of CD81, Aβ42, Tau and p-Tau T231 were measured using a standard ELISA kit (CD81, Cusabio) or Simoa technology on the SR-X instrument (Quanterix, MA). Importantly, the levels of AD-associated biomarkers were higher in the DS-AD NDEVs than in the control NDEVs (Table 1), supporting the morphological results presented below.

### 3.2. Tau Protein Amplification and Aggregate Structure

A highly sensitive assay that can amplify minute quantities of fibrillar seeds using consecutive cycles of shear-induced fragmentation [24,25,26,88] was utilized to determine the seeding capacity of the NDEVs (Figure 2D). The amplification efficiency depends on the original amounts of aggregates and their conformations. This assay was successfully applied to an AD brain extract previously [24] and was able to amplify seeding competent p-Tau from the NDEVs purified from patients with DS-AD in the current study (Figure 2D). Importantly, no Tau amplification was observed for the control NDEVs, indicating that DS NDEVs contain seeding competent Tau aggregates as a proof of principle for the in vivo component of this study. These data strongly suggest that the NDEVs used in the current study represented exosomes released from Tau-bearing neurons in the CNS, containing structural abnormalities of Tau that gave rise to its seeding competence.

### 3.3. Increased p-Tau Staining Intensity 4 Months after the Injection 

**Figure 4:**
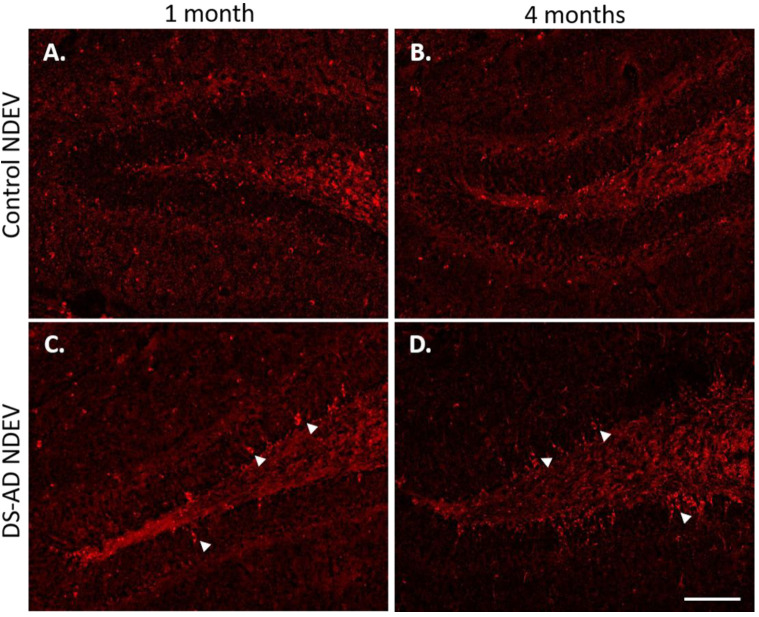
Representative images of p-Tau (S396) staining in WT mouse dentate gyrus area 1 month or 4 months following intra-hippocampal injections of NDEVs enriched from plasma from a control case (**A**,**B**) and a DS-AD case (**C**,**D**). Note the significant increase in p-Tau inclusions in neurons after DS-AD NDEV injections (arrowheads in (**C**,**D**)), especially in neurons located in the granule cell layer (GCL). Few, if any, intraneuronal p-Tau inclusions were observed in brains injected with control NDEVs (**A**,**B**). Scale bar in (**D**) represents 100 μm.

**Figure 5:**
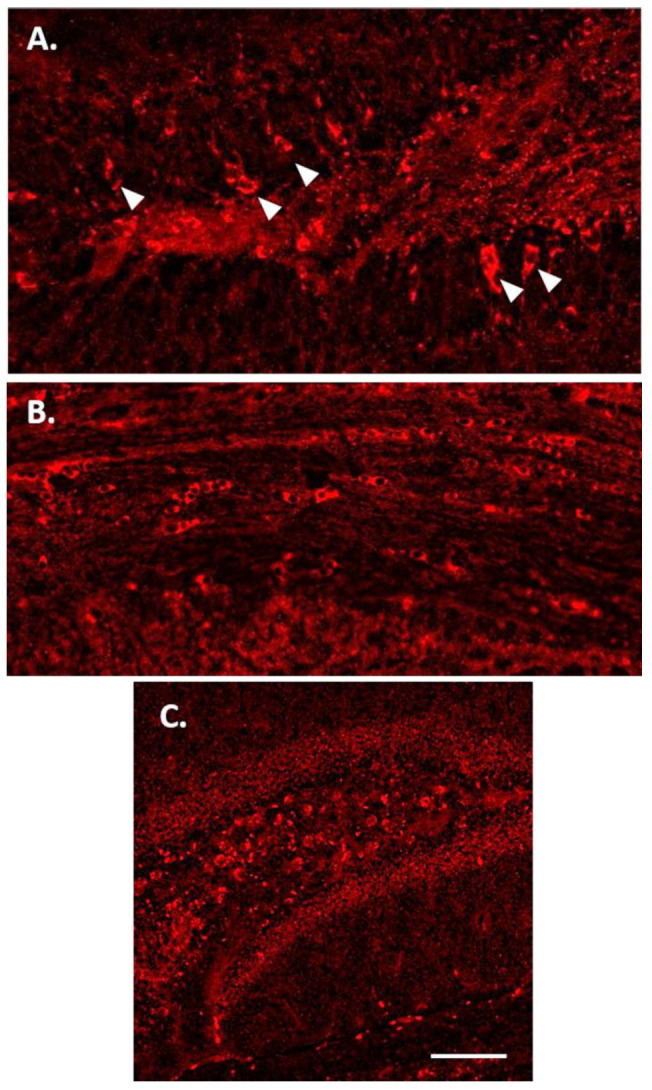
p-Tau immunostaining 4 months after injection of DS-AD NDEVs. There was a wide distribution of p-Tau S396-positive cells within the GCL of the dentate gyrus (**A**) and the corpus callosum (**B**). (**C**) Representative p-Tau T231 immunostaining in the hippocampus 1 month following DS-AD NDEV injection. Note the difference in distribution, with large neurons in the hilar region staining in a uniform pattern without strong “flame-like” tangle staining in the apical dendrite. Scale bar in C represents 100 microns.

To determine whether the injection of NDEVs from DS-AD or the controls gave rise to the increased p-Tau staining in the adult mouse hippocampus, two different p-Tau antibodies were used to stain for the T231 and S396 p-Tau epitopes, respectively. While the S396 antibody used here mostly binds mature NFTs, the T231 antibody binds pre-tangle Tau pathology [21,83]. We found a significant increase in p-Tau staining intensity in the dentate of the mice injected with DS-AD NDEVs, compared to the controls (see Figure 4), and this staining was even more prominent 4 months post-injection in DS-AD vs. Control NDEVs-injected WT mice. The p-Tau S396 staining appeared to be mostly neuronal and included flame-like apical morphology reminiscent of NFT pathology (see arrows on Figure 5A). In the dentate formation, the p-Tau S396 immunostaining was mostly present in the granule cell layer and at the interface between the granule cell layer and the polymorphic layer of the dentate gyrus (Figure 4C,D). Immuno-positive cells could also be observed in small cells along the corpus callosum (Figure 5B), indicating a spread to other brain regions possibly via glial cell migration. The distribution of the p-Tau T231 immunostaining was notably different from that seen for the p-Tau S396 antibody and did not show flame-like inclusions in the DG GCL (Figure 5C). Instead, intracellular staining could be observed inside the entire cytoplasm in large hilar neurons, with minimal staining observed in the granule cell layer (Figure 5C).

**Figure 6:**
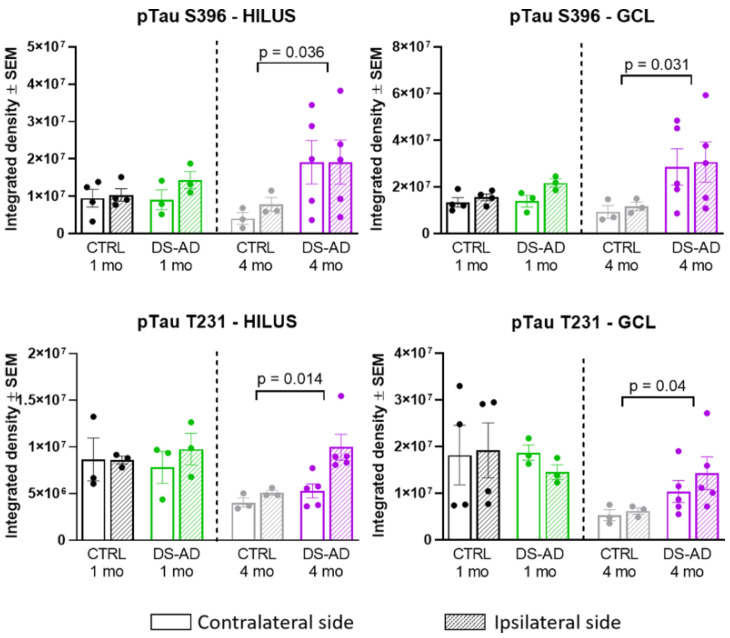
Densitometry of p-Tau S396, and p-Tau T231 in the Hilar region (**left panels**) and the GCL (**right panels**) of the hippocampus. There was a significant increase in p-Tau S396 staining in the Hilar region between DS-AD and controls at 4 months (*p* = 0.03) and in the GCL (*p* = 0.03). In addition, there was a significant increase in T231 p-Tau after 4 months in mice injected with DS-AD vs. Controls in the hilar region (*p* = 0.014) and in the GCL (*p* = 0.04).

A two-way ANOVA on p-Tau S396 densitometry measurements (Figure 6, upper part) showed significant effects of the treatment group 4 months after the injection of the NDEVs in the hilar region (F_1,12_ = 5.55, *p* = 0.036) as well as in the granule cell layer of the DG (GCL; F_1,12_ = 5.945, *p* = 0.031). Similarly, the p-Tau T231 densitometry measurements (Figure 6, lower part) were significantly different between the treatment groups in the hilar region (F_1,12_ = 8.172, *p* = 0.014) as well as in the granule cell layer of the DG (F_1,12_ = 5.295, *p* = 0.040) 4 months after the injection of the NDEVs. Overall, our data show that four months was a sufficient time to result in higher p-Tau S396 and T231 staining intensities in the dentate gyrus of mice injected intra-hippocampally with NDEVs obtained from DS-AS blood compared to mice injected with control NDEVs. In addition, numerous S396-stained inclusions were observed along the GCL in DS-AD but not in control-NDEV injected mice at 1 month post-injection (Figure 4A,C), suggesting rapid conformational changes of Tau in the WT mice injected with human NDEVs from persons with DS-AD—further supported by the data presented in Figure 2D.

### 3.4. Glial Immunostaining

**Figure 7:**
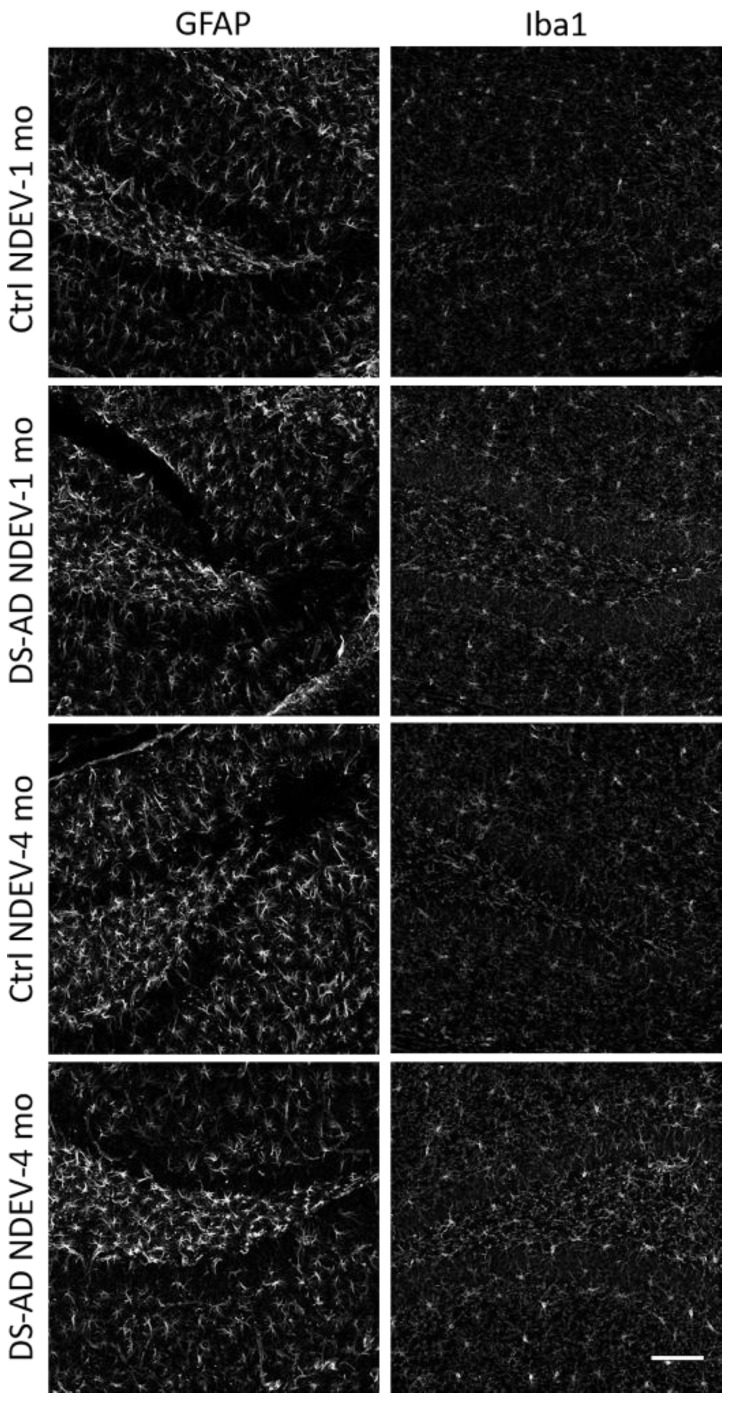
Representative images for GFAP (on the left) and Iba1 (on the right) immunofluorescent staining. Note an increase in staining intensities in mice who received DS-AD NDEVs compared to control NDEV injections. The astrogliosis following DS-AD NDEVs was more obvious but also displayed more variability between animals 4 months following the injections. Note an increase in Iba1 staining intensity in DS-AD NDEV-injected mice 4 months following injection, and to a lesser extent 1 month following injection. Scale bar represents 100 microns.

**Figure 8:**
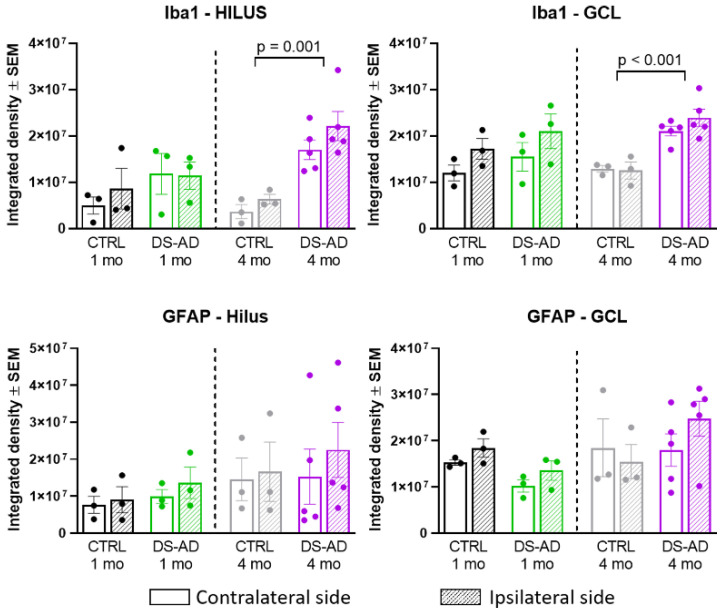
Densitometry for Iba1 and GFAP showed increased glial activation in mice injected with the DS-AD NDEVs compared to controls. Density measurements for Iba1, but not GFAP, were significantly elevated 4 months after DS-AD injection compared to controls (*p* < 0.001).

To determine whether the NDEVs from controls or DS-AD gave rise to long-lasting neuroinflammation in the recipient injected brain region, we examined staining density for GFAP and Iba1 (Figure 7) 1 or 4 months following the intrahippocampal injection. Iba1 immunohistochemistry showed significant microglia activation in mice injected with DS-AD NDEVs, compared to those injected with NDEVs from control blood (Figure 7). This was observed to a much greater extent 4 months after injection, compared to 1 month post-injection (Figure 7). A similar increase staining of astrocytes could also be observed using the astrocyte marker GFAP (Figure 7), although this reaction to the NDEV injections was variable between animals and was not as robust as the microglial activation observed with Iba1. However, densitometry measurements were conducted on the Iba1 and GFAP staining density in the Hilar region as well as in the GCL (Figure 8). A significant increase in the Iba1 staining intensity could be observed in both these sub-regions of the hippocampus between control- and DS-AD-injected mice (*p* ≤ 0.001) 4 months after the injection (Figure 8), but not after 1 month. However, the densitometry of GFAP labeling revealed a more variable effect of the NDEV injections, with increased gliosis observed also in some control-NDEV injected mice (Figure 8, lower part).

**Figure 9:**
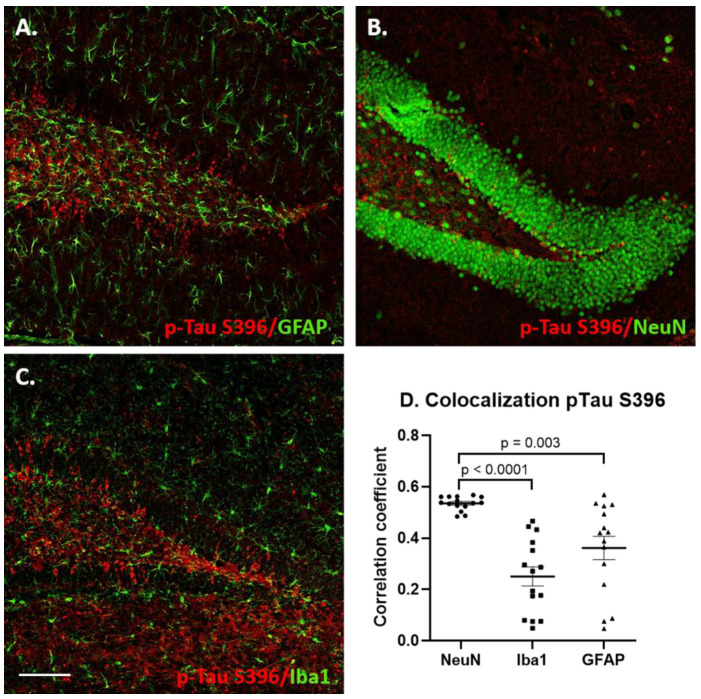
Co-localization of p-Tau S396 staining with neuronal and glial markers in the dentate gyrus region of hippocampus 1 month following DS-AD NDEV injections. (**A**) Double labeling for p-Tau S396 (red) and GFAP (green), (**B**) double labeling for p-Tau S396 (red) and NeuN (green), (**C**) double labeling for p-Tau S396 (red) and Iba1 (green). Scale bar in C corresponds to 100 microns. The scatter plot graph (**D**) shows the correlation coefficients for p-Tau S396 with NeuN, Iba1 and GFAP.

**Figure 10:**
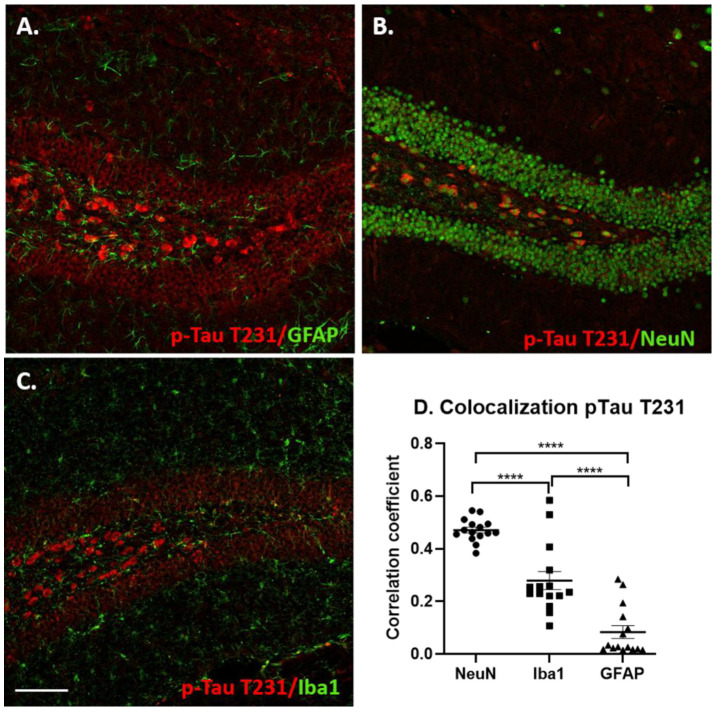
Co-localization of p-Tau T231 staining with neuronal and glial markers. (**A**) Double labeling for p-Tau T231 (red) and GFAP (green), (**B**) double labeling for p-Tau T231 (red) and NeuN (green), (**C**) double labeling for p-Tau T231 (red) and Iba1 (green). Evaluation of the double-labeled sections revealed p-Tau T231 staining was largely located to neurons, with some staining observed in microglia (Iba1) and to a lesser extent astrocytes (GFAP). The scatter plot graph (**D**) shows the correlation coefficients for p-Tau T231 colocalization with NeuN, Iba1 and GFAP (****, *p* < 0.0001).

To determine which cell types contained p-Tau-immunoreactive cells following the NDEV injections, immunofluorescent double labeling was conducted with NeuN (neuronal marker), GFAP (astrocyte marker) and Iba1 (microglial marker), see Figure 9, Figure 10, Figures S1 and S2. When the markers were analyzed for co-localization, there were differences in the p-Tau presence in glial cells between the two epitopes. For the p-Tau S396 antibody, most staining was localized in NeuN-immunopositive neurons, compared with GFAP (*p* = 0.003) and Iba1 (*p* < 0.001) staining, although some astrocytes and microglial cells containing the S396 epitope antibody could also be observed (Figure 9). Most p-Tau/NeuN double-labeled neurons were observed in the granule cell layer, with some scattered neurons seen in the hilar region of the dentate gyrus (Figure 9). Pixel intensity correlation confirmed these findings (Figure 9D). However, it could be seen that some astrocytes also contained staining for this p-Tau epitope. Previous studies have shown that p-Tau inclusions can appear in astrocytes in aggressive AD pathology and contribute to neuroinflammation and to the spread of pathology [89].

As mentioned above, the distribution of p-Tau T231 immunoreactivity was significantly different from that of the staining for the S396 p-Tau epitope. P-Tau T231-positive cells were mostly observed in the hilar region of the dentate gyrus and in CA4/CA3 (not shown). Figure 10 shows the double labeling of p-Tau T231 with GFAP (A), NeuN (B), Iba1 (C). This epitope was almost exclusively located within neurons and an intensity correlation for co-localization confirmed this observation, demonstrating a highly significant percentage of neurons co-labeled with NeuN compared to both Iba1 (*p* < 0.001) and GFAP (*p* < 0.001) immunostained cells (Figure 10D).

## 4. Discussion

The findings presented here demonstrate that the NDEVs derived from the plasma of persons with DS and AD gave rise to an accumulation of both the T231 and S396 forms of p-Tau when injected into the adult mouse hippocampus. The p-Tau inclusions were more evident in the 4-month versus the 1-month post-injection mice, as determined using densitometry, and was significantly more abundant after an injection of DS-AD NDEVs compared to the control NDEVs. Prior to the injection of the NDEVs into the mouse brain, we also demonstrated that the NDEVs had the requisite exosome-related tetraspanin markers, that DS-AD NDEVs were able to elicit the amplification of seeding competent p-Tau, and that the average particle size was comparable to what has been reported for exosomes in the literature [90]. The current findings suggest that despite the relatively low levels of p-Tau in exosomes compared to the levels recovered from AD brain tissue, the p-Tau within the NDEVs could induce the templated misfolding of endogenous murine Tau and thereby transfer Tau pathology from the human samples to the mouse brain, wherein p-Tau pathology was propagated to un-injected areas. 

In addition to the observed p-Tau species intra-neuronally, DS-AD NDEVs also gave rise to a significant activation of both microglia and, to a lesser extent, to astrocytes in the hippocampus. These findings suggest that small neuron-derived EVs from persons with DS-AD can propagate both p-Tau pathology and neuroinflammation when injected to another species and further supports the notion that neuron-derived extracellular vesicles may be heavily involved in the spreading of AD pathology in the brain. 

Previous studies by others and by our group have shown that individuals with DS have an increased release of exosomes [91,92], potentially due to the diminished capacity of the lysosomal/autophagosome system in DS [93,94,95]. Gauthier and colleagues [91] suggested that an increased expression of the CD63 tetraspanin enhances exosome release in DS as a protective mechanism to counteract the endosomal abnormalities and the toxic buildup of Tau and amyloid peptides in the DS brain. It is known that cellular oxidative stress can increase exosome release [96,97], while stimulation of autophagy using the mTOR inhibitor rapamycin greatly inhibits exosome release [98]. Therefore, it is possible that the mTOR dysregulation observed in those with DS [93,99] contributes to the deficiencies in the autophagy system and hence causes an increased exosome release, as suggested by others [98]. We have also previously shown that NDEVs derived from DS plasma contain high levels of both amyloid and p-Tau—already in children with DS [13]. The current study is an extension of this previous work, showing that p-Tau in the NDEVs is seeding-competent and can be propagated to neurons and glia in another species. 

Significant dysfunction of the endosomal pathway along with abnormally enlarged early endosomes have been reported to be early characteristics of AD—both in the general population and in DS [94,100,101]. Endosomal material is packaged intracellularly into MVBs and released by several different mechanisms into the extracellular space via exosomes. CD63 knockdown gives rise to diminished exosome secretion and, therefore, also increases the intracellular pathology in DS fibroblasts [91]. Based on these findings, the stimulation of exosome release may appear as a novel promising therapeutic alternative for both DS and AD. However, based on the results presented herein, it could also contribute significantly to spreading AD pathology. 

An important aspect of AD pathology is the mechanisms by which both Tau and amyloid pathology spreads from one brain region to another and eventually envelopes most brain regions [15]. The progressive aggregation of misfolded hyperphosphorylated forms of Tau is one of the two important hallmarks for the disease. The misfolded forms of p-Tau spread from the entorhinal cortex to the hippocampus early on in AD [102], and from there on to the frontal cortex and other brain regions. Recent findings suggest that there are at least the following three different ways in which p-Tau can spread from neurons to other neurons or glia: (A) translocation through the plasma membrane; (B) membranous organelle-based secretion; and (C) ectosomal shedding [15]. Glial cells have also been shown to contribute to the propagation of Tau pathology [103]. These previous findings fit with our current findings, showing that at least some glial cells contained p-Tau inclusions following an injection of the DS-AD NDEVs.

Our data herein indicate that Tau can also spread via exosomal delivery—something that has been shown for exosomes purified from AD patients previously [68,70,71,104] but has not been shown for exosomes derived from DS or DS-AD. Others have shown that injecting with human seeding competent p-Tau can give rise to an alteration of the murine Tau metabolism and thus lead to both the production and aggregation of 3R-Tau and 4R-Tau [36]. This is thought to occur via the activation of Tau kinases in the mouse recipient cells. Our findings suggest that the delivery of toxic p-Tau via NDEVs can lead to a similar process, since staining for the two p-Tau phosphorylated forms used here (T231 and S396) is very low to absent in non-injected mice, at least at the age and strain used here. In addition, the intracranial delivery of NDEVs from control participants did not elicit this strong response in terms of p-Tau staining. This is further supported by the fact that p-Tau in the NDEVs from DS-AD could be amplified but this was not seen for control NDEVs, at least not in our hands (Figure 2D). An important contributor to Tau propagation also appears to be neuronal activity, but these findings are preliminary and remain to be investigated further [105]. In the brain, the uptake of exosomes into neurons occurs either via phagocytosis or endocytosis [54], while microglial cells take up exosomes via micropinocytosis [106]. Ruan and collaborators suggested that exosome-delivered p-Tau preferentially spread to the hippocampal GABA-ergic interneurons when the AD-exosomes were injected into the mouse hippocampus [71]—this finding remains to be investigated for DS-AD-derived NDEVs and will be the topic of future investigations. It was interesting to note that in our current study, astrocytes—and to some extent microglia—contained the S396 form of p-Tau but not the earlier and less aggregating T231 form of p-Tau. 

Tau-inclusions have been reported in astrocytes in the hilar region of the hippocampus in AD [89]. These investigators reported that astrocytic p-Tau affected mitochondrial function and led to the loss of inhibitory neurons in the hippocampus [89]. In the current study, there was a profound glial reaction following the DS-AD NDEVs but not the control NDEVs injection—this glial reaction appeared to worsen with time, since densitometry showed increased Iba1 and GFAP staining in the 4-month compared to the 1-month groups. This could be due to either pro-inflammatory cytokines delivered via the NDEV cargo [107,108] or a reaction to the Tau or amyloid species in the cargo, since both Tau and amyloid can elicit an inflammatory response [109,110,111]. It has been shown that NDEVs can be taken up not only by neurons but also by glial cells [112]. You and collaborators [113] showed that the exosomes that were isolated from IL-1β-stimulated astrocytes contained increased levels of integrins—which increased the ability of the exosomes to be taken up by receptor cells. Although this is a likely pathway to increased uptake, we do not know yet if this is a feature of DS-AD NDEVs as well and this will also be a focus of continued studies. Those with DS have a dysregulation of their innate immune system and it is possible that an inflammatory profile of the cells of origin can spread to the recipient cell either via cytokines, integrins or miRNAs [114,115,116,117,118,119] and, therefore, increase the propagation of toxic Tau species within the brain. As an example of exosome-mediated inflammation “spread”, Pascual and collaborators demonstrated that in an experimental mouse model of autoimmune encephalomyelitis, pro-inflammatory cytokines promoted the release of exosomes by immune cells, which induced further pro-inflammatory molecules by spreading the inflammation to the recipient cells [112]. Exosomes are known to transport misfolded pathogenic proteins or dysregulated miRNAs into neurons and glial cells, which can then act to both initiate and propagate neuroinflammation in the target cells [108]. Interestingly, at least five miRNAs have been identified on chromosome 21, of which two or more are directly involved in inflammation [114,120,121,122,123]. Thus, a natural next step in these studies will be to identify not only the protein but also the miRNA cargo in DS-AD NDEVs to determine their role in the propagation of inflammation from NDEVs, as observed in the current study. EV cargo can contain cytokines, including IL-1β, IL-10, eotaxin and TNF-α [124]—each of these cytokines can initiate and propagate inflammation in recipient cells. Similar to the plasma from persons with DS, the blood from persons with autism spectrum disorder (ASD) contains increased amounts of EVs, and these can stimulate microglia in vitro to produce increased levels of IL-1β, thus propagating a pro-inflammatory profile [125]. Recent evidence suggests that EVs secreted in the brain contain inflammatory mediators that can communicate with neurons, thus contributing to the pathogenesis and propagation of neuroinflammation [112]. Our findings herein suggest that intracranial injections of NDEVs isolated from individuals with DS-AD can elicit a neuroinflammatory response in the vicinity of the injection site, which may lead to both the propagation of neuroinflammation and the spread of toxic Tau forms. 

Our findings, and those by others, suggest that both amyloid-beta and p-Tau pathology can spread from one individual to another or from one cell to another cell via exosomes [65,67,68,70,104,108,126,127,128]. This is a notable finding, since EVs can be purified from all body fluids including urine, plasma, serum and saliva [90,129]. Although we did not find evidence of observable amyloid propagation from the DS-AD NDEVs, Prusiner and colleagues have suggested that AD is a double-prion disorder. This group has demonstrated the seeding-competent species of both amyloid and Tau prions in *postmortem* brain samples, with the highest levels observed in early onset-AD cases [130]. This is the first study that shows that the NDEVs isolated from DS-AD blood contain aggregation-prone Tau species that can spread within the brain after an intracranial injection. Work will now continue to further examine the extent of p-Tau spread within the mouse brain at different post-injection times, which neuronal populations are affected by NDEV-derived p-Tau and whether amyloid-beta can be detected in the mouse brain following NDEV injections. 

## 5. Conclusions

There is no doubt that exosomes impart important functions in the brain during normal physiological conditions including trophic support, synaptic activity and neuronal survival. However, in pathological circumstances caused by either environmental or genetic conditions, exosomes can contribute to the spread of pathology, as well as implement and propagate pathological agents and neuroinflammation. Our findings here suggest that NDEVs purified from persons with DS and AD can solicit p-Tau neuronal and glial inclusions in mice as well as give rise to the significant activation of glial components in the area of injection. Work in this area will now continue to identify the specific cargo components that are responsible for the pathology observed. This will include both proteins and miRNA as well as other components delivered via exosomes and can give important information regarding the spread of AD pathology within the brain of one individual or between individuals as well as provide targets for drug development in the future to prevent spreading. There are FDA-approved drugs that affect the biogenesis, release and uptake of exosomes [131] that have been employed in the cancer field but that could be repurposed for successful treatment of AD as well. A recent study demonstrated that inhibiting exosome synthesis in a mouse model reduced the spread of toxic Tau species from the entorhinal cortex to the hippocampus [102], suggesting that this approach may be worth trying. Studies of exosomes and their biogenesis, release and uptake mechanisms may, therefore, provide a novel mechanistic view of the disease phenotype.

## Figures and Tables

**Table 1:** Levels of biomarkers measured in NDEV enriched from plasma samples from one participant with DS-AD and one control.

	Control NDEV	DS-AD NDEV
CD81 (ng/mL)	15.21	23.51
Aβ42 (pg/mL)	<LOD	31.98
Tau (pg/mL)	23.12	35.38
p-Tau T231 (pg/mL)	57.01	242.26

NDEV: neuron-derived small extracellular vesicle; DS-AD: Down syndrome exhibit Alzheimer’s Disease; LOD: limit of detection.

## Data Availability

The data presented in this study are available from the corresponding author on reasonable request.

## Supplementary Materials

The following are available online at https://www.mdpi.com/article/10.3390/jcm10173931/s1, Figure S1: Unmerged confocal images (from representative images shown in Figure 9) of p-Tau S396 staining ((A,C,E) red) with corresponding double labelling ((B): GFAP, (D): NeuN and (F): Iba1, green). Scale bar in E corresponds to 100 microns, Figure S2: Unmerged confocal images (from representative images shown in Figure 10) of p-Tau T231 staining ((A,C,E) red) with corresponding double labelling ((B): GFAP, (D): NeuN and (F): Iba1, green). Scale bar in E corresponds to 100 microns.
