# Supplementary material for: Small Neuron-Derived Extracellular Vesicles from Individuals with Down Syndrome Propagate Tau Pathology in the Wildtype Mouse Brain
Source: J Clin Med. 2021 Aug 31;10(17):3931. doi: 10.3390/jcm10173931 (PMC8432237; doi:10.3390/jcm10173931)
Supplement: Supplementary file 1 [file jcm-10-03931-s001.zip › jcm-1344527-supplementary.pdf]

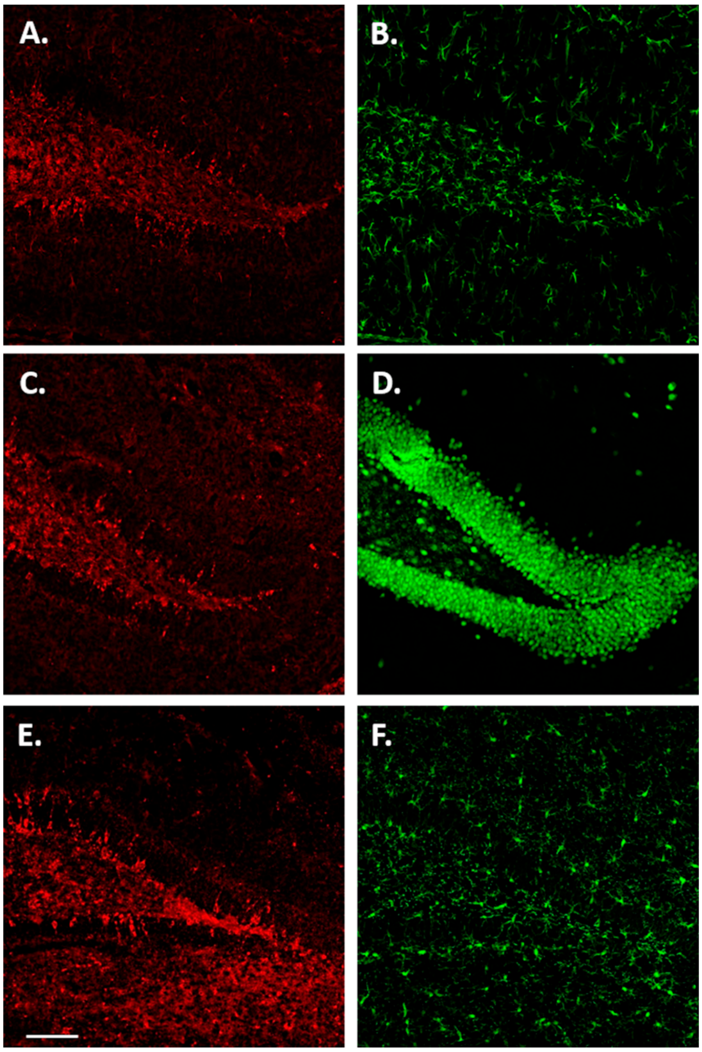

Figure S1. Unmerged confocal images (from representative images shown in Figure 9) of p-Tau S396 staining ((**A**,**C**,**E**) red) with corresponding double labelling ((**B**): GFAP, (**D**): NeuN and (**F**): Iba1, green). Scale bar in E corresponds to 100 microns.

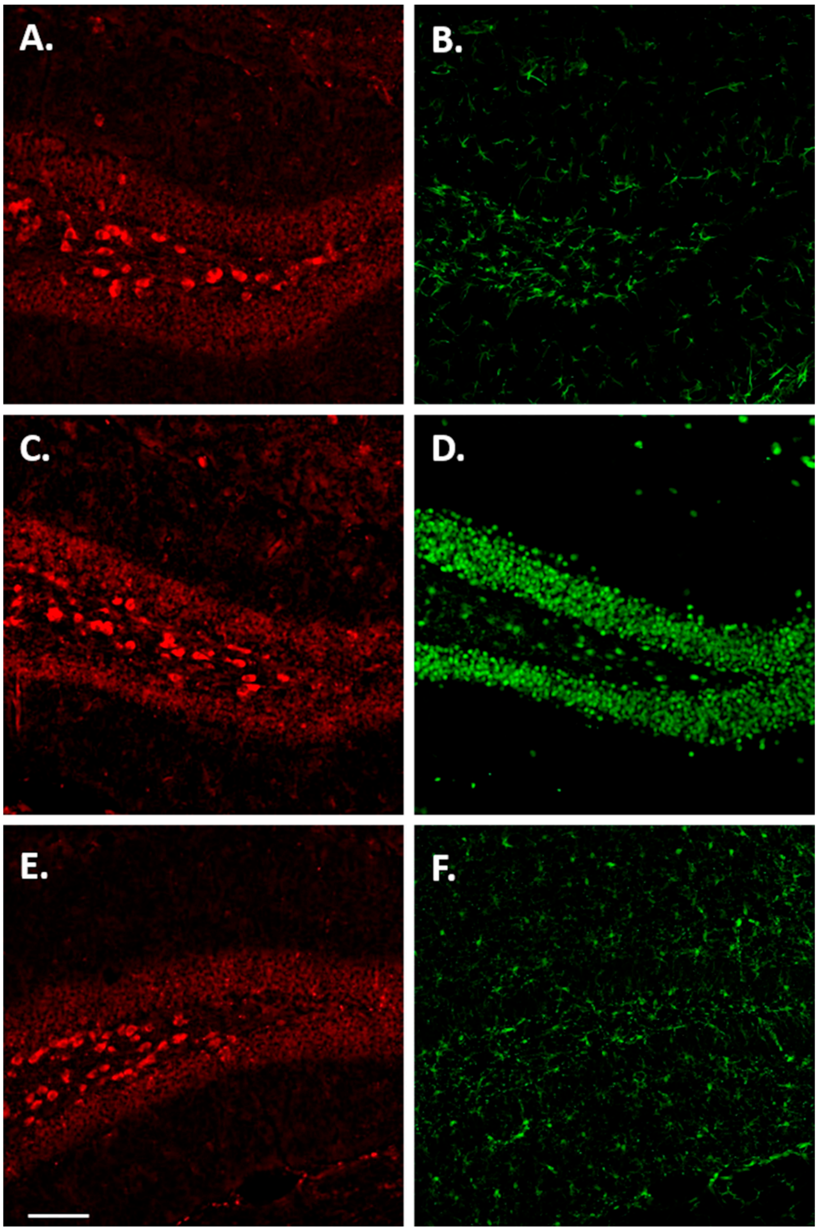

Figure S2. Unmerged confocal images (from representative images shown in Figure 10) of p-Tau T231 staining ((**A**,**C**,**E**) red) with corresponding double labelling ((**B**): GFAP, (**D**): NeuN and (**F**): Iba1, green). Scale bar in E corresponds to 100 microns.
